# Posterior reversible encephalopathy syndrome (PRES) induced by pazopanib, a multi-targeting tyrosine kinase inhibitor, in a patient with soft-tissue sarcoma: case report and review of the literature

**DOI:** 10.1007/s10637-017-0521-5

**Published:** 2017-10-25

**Authors:** Shoichi Deguchi, Koichi Mitsuya, Yoko Nakasu, Nakamasa Hayashi, Hirohisa Katagiri, Hideki Murata, Junji Wasa, Mitsuru Takahashi, Masahiro Endo

**Affiliations:** 1 Divisions of Neurosurgery, Shizuoka Cancer Center, 1007, Shimo-nagakubo, Naga-izumi, Shizuoka, 411-8777 Japan; 2 Orthopedic Oncology, Shizuoka Cancer Center, Shizuoka, Japan; 3 Diagnostic Radiology, Shizuoka Cancer Center, Shizuoka, Japan

**Keywords:** Posterior reversible encephalopathy syndrome, Soft-tissue sarcoma, MRI, Pazopanib

## Abstract

Posterior reversible encephalopathy syndrome (PRES) is a clinical entity characterized by acute neurological symptoms such as severe headache, seizures, and visual disturbance, and by typical reversible lesion on brain magnetic resonance (MR) images. Since PRES is thought to be caused by vascular endothelial injury due to cytotoxic agents or acute systemic hypertension, the number of reports on PRES associated with angiogenesis inhibitors has been increasing. Although five cases that developed PRES due to pazopanib for renal cell carcinoma have already been reported, none of PRES due to pazopanib for soft-tissue sarcoma has been reported thus far. We describe a case of a 49-year-old woman with retroperitoneal soft-tissue sarcoma who developed PRES during pazopanib administration. Pazopanib at 800 mg/day was administered as her third-line treatment at relapse. After 38 days of pazopanib, she was admitted to our hospital with severe headache, vomiting, and systemic hypertension. The next day, she developed consciousness deterioration and visual disturbance together with exacerbated systemic hypertension. Brain MR images revealed hyper-intense signals on FLAIR sequences in the bilateral occipital lobes and the left thalamus. Intravenous nicardipine injection was immediately started to control her blood pressure and pazopanib was discontinued. Her symptoms gradually improved and disappeared on the fifth hospital day. After 2 weeks, hyper-intense signals on a FLAIR sequence disappeared completely. She restarted a low dose of pazopanib under good blood pressure control and experienced no subsequent recurrence of PRES.

## Introduction

Posterior reversible encephalopathy syndrome (PRES) is a clinical entity characterized by acute neurological symptoms such as severe headache, seizures, and visual disturbance, and by reversible lesion hyper-intensity on FLAIR and T2-weighted sequences on brain magnetic resonance (MR) images, especially in the bilateral occipital and parietal lobes [1, 2]. PRES is thought to arise from vascular endothelial injury due to cytotoxic agents or acute systemic hypertension [2]. Recently, there has been an increase in the number of reports about PRES associated with not only conventional chemotherapeutic agents, but also new molecular targeted drugs, particularly angiogenesis inhibitors, which may cause systemic hypertension as an adverse effect [3, 4]. Pazopanib is an oral tyrosine kinase inhibitor that targets vascular endothelial growth factor receptor, platelet-derived growth factor receptor, and c-Kit, and is approved for use in advanced renal cell carcinoma and soft-tissue sarcoma [5–7]. Five cases of PRES induced by pazopanib for renal cell carcinoma have been reported [8–12]. However, no case of PRES due to pazopanib for soft-tissue sarcoma has been reported in the English literature. Here, we present a patient of this type and describe her clinical course.

## Case report

**Fig. 1:**
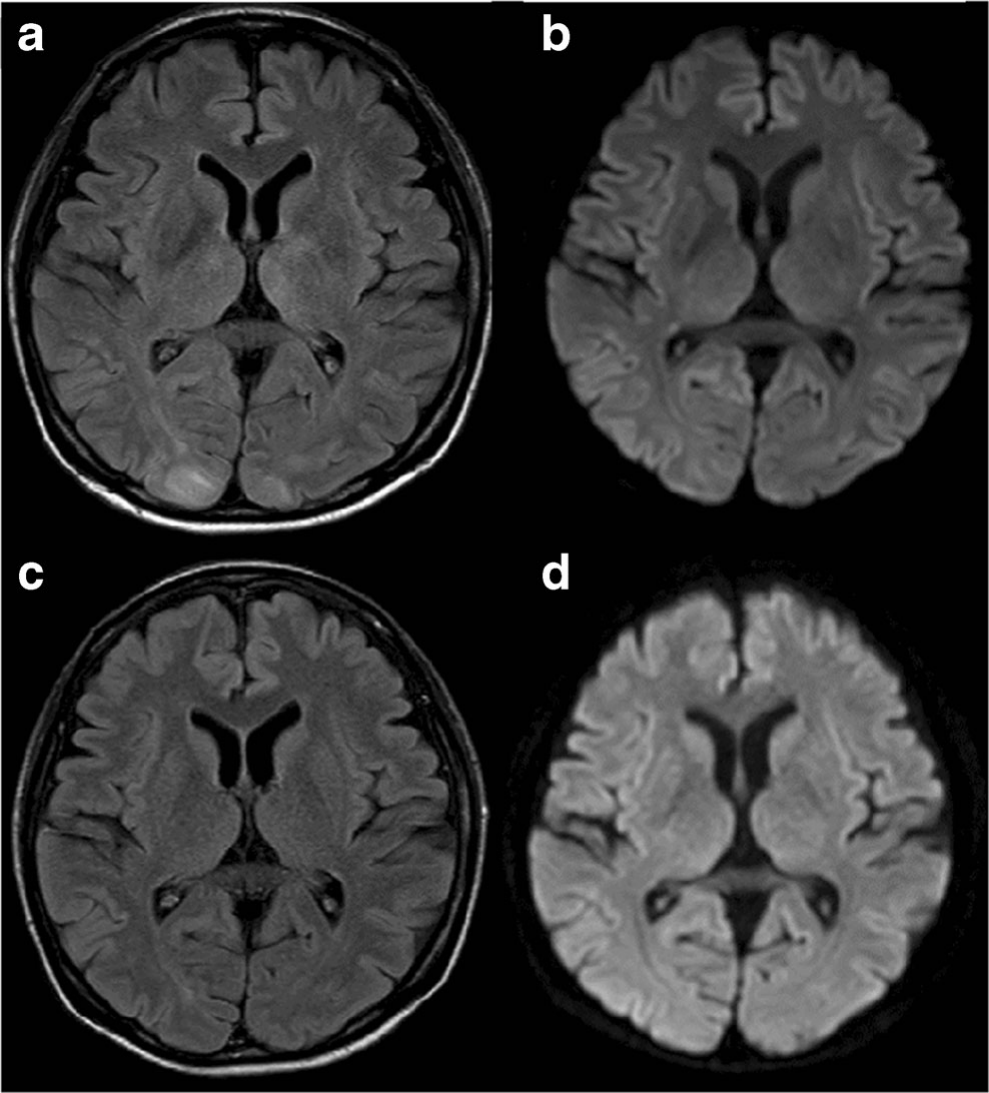
FLAIR (**a**) and DWI (**b**) on brain MR images at onset of PRES. FLAIR (**c**) and DWI (**d**) on brain MR images of the same patient after 3 weeks

We report a 49-year-old woman with retroperitoneal soft-tissue sarcoma. She had no previous history of systemic hypertension, renal dysfunction, or autoimmune disease. She was diagnosed by computed tomography (CT)-guided needle biopsy of the tumor. Gemcitabine and docetaxel were administered as initial treatment, but the disease progressed after five cycles of chemotherapy. Ifosfamide and adriamycin were administered as a second-line treatment for 8 months, achieving partial response as a best response, but were stopped due to severe and prolonged myelosuppression. Three months later, pazopanib at 800 mg/day was administered as a third-line treatment at relapse. She had taken fentanyl transdermally and a sublingual tablet as pain relief, along with proton pump inhibitors, sleep inducers, and an anti-emetic drug on a regular basis. Twenty-eight days later, the dose of pazopanib was reduced from 800 to 600 mg/day because of nausea and anorexia. Another two days later, CT examination revealed that she had achieved a partial response. However, another eight days later, she was admitted to our hospital with severe headache, nausea, and vomiting. Her blood pressure was 154/87 mmHg on admission. Blood tests including complete blood count, blood chemistry, and coagulation showed no abnormal findings other than hyponatremia (129 mEq/L). Renal function was in the normal range (estimated GFR: 70.4 ml/min/1.73m^2^). Urine analysis showed no proteinuria. Brain MR images without contrast agent showed normal findings. Supportive care did not improve her symptoms. The next day, she suddenly presented consciousness deterioration (Glasgow Coma Scale 13 points) and visual disturbance along with severe systemic hypertension (201/108 mmHg). Brain MR images revealed hyper-intense signals in the bilateral occipital lobes and left thalamus on FLAIR sequences, but no lesions on a diffusion-weighted image (DWI) (Fig. 1). Under a diagnosis of PRES, she was treated with anti-convulsant (fosphenytoin sodium hydrate at 375 mg/day), betamethasone (4 mg/day), osmotic diuretics (glycerol at 200 ml/day), and anti-hypertensive drug (nicardipine, continuous intravenous injection). Her systolic blood pressure could be controlled below 130 mmHg. At the same time, pazopanib was discontinued. Her symptoms gradually improved and disappeared on the fifth hospital day. She was discharged on the seventh hospital day. Two weeks later, brain MR images revealed complete disappearance of the hyper-intense signals on FLAIR sequence (Fig. 1). Then, oral administration of pazopanib at 400 mg/day was again applied along with the anti-hypertensive drug. Ultimately, the dose was escalated to 600 mg/day and there was no recurrence of PRES under good blood pressure control. Unfortunately, the patient died 9 months after restarting pazopanib because of progression of the primary tumor.

## Discussion

Soft-tissue sarcomas are rare malignant tumors derived from mesodermal tissues such as skeletal muscle, adipose, and fibrous tissue [13]. Pazopanib was approved for treatment of metastatic soft-tissue sarcomas by the US Food and Drug Administration based on a phase III trial (PALETTE) in 2012. In the PALETTE study, pazopanib significantly improved the progression-free survival (PFS) of patients with soft-tissue sarcomas. On the other hand, it caused systemic hypertension as an adverse event in 41% of the patients (CTCAE Grade 3: 7%) [7]. Acute and severe systemic hypertension may cause vasodilation and disruption of cerebral autoregulation, resulting in breakdown of the blood–brain barrier [2]. In addition, pazopanib inhibits the VEGF and PDGF pathway, which may lead to vascular endothelial damage [14]. These pathophysiological changes might cause vascular leaks and cerebral edema, resulting in PRES due to pazopanib. The case reports of PRES induced by pazopanib are compiled in Table 1 [8–12]. All patients developed PRES with marked systemic hypertension after more than a week of pazopanib administration. Brain edema occurred mostly in bilateral occipital lobes, not in cerebellum or brain stem. Most patients recovered within a week by withdrawal of pazopanib and anti-hypertension therapy. These features are consistent with our case of soft-tissue sarcoma.

**Table 1:** Characteristics of patients with PRES induced by pazopanib

Authors	Primary tumor	Age (years)	Sex	Dose of pazopanib	Duration from onset of PRES to starting pazopanib	Symptoms	Location	BP at PRES presentation	Treatment	Outcome	Time to resolution
Chelis et al. [8]	RCC	40	F	800 mg/d	21 days	headache, seizure, vision disturbance	bi-frontal, parietal, occipital lobes	165/105	phenytoin, mannitol, antihypertensive agents	resolved	1 day
Foerster et al. [9]	RCC	62	F	800 mg/d	2 months	headache, seizure, left arm paresis, gait instability, nausea, vomiting	left parietal lobe	> 300	diazepam, levetiracetam, anti-hypertensive agents	resolved	6 days
Asaithambi et al. [10]	RCC	76	M	800 mg/d	1 month	headache, vision disturbance, vomiting, disorientation	bi-temporal, parietal, occipital lobes	219/155	anti-hypertensive agents	resolved	2 days
Miaris et al. (11)	RCC	56	F	800 mg/d	9 days	headache, nausea, vomiting, dizziness, gait instability	bi-frontal, parietal, occipital lobes	165/ 95	levetiracetam, antihypertensive agents	resolved	3 days
Miller-Patterson et al. [12]	RCC	69	F	not shown	3 weeks	headache, left-sided weakness	bi-occipital lobes (combined hemorrhage)	204/92	anti-hypertensive agents	resolved	not shown
Present case (2017)	Softtissue sarcoma	52	F	800 mg/d	38 days	headache, nausea, seizure, vision disturbance	bi-occipital lobes, left thalamus	201/108	osmotic diuretics, betamethasone, fosphenytoin, antihypertensive agents	resolved	5 days

PRES is not always completely reversible and its associated mortality rate has been reported to be about 3%–6% [2]. Elimination of the causative factors at an early stage is critical to prevent poor outcomes [2]. Further, in cases of PRES associated with systemic hypertension, a general consensus has been reached about the importance of prompt anti-hypertension therapy. An initial goal of reducing blood pressure by 25% within several hours of the onset of PRES using continuous intravenous administration of anti-hypertensive agents should be set [15]. In fact, in our case as well, the neurological deficits dramatically improved with the control of blood pressure, suggesting the efficacy of anti-hypertensive therapy for PRES.

As for another angiogenesis inhibitor, bevacizumab (BV), sub-analysis of a phase III trial of BV plus interferon alfa versus interferon alfa monotherapy in patients with metastatic renal cell carcinoma showed that patients who developed systemic hypertension by BV exhibited significant improvements in PFS and overall survival compared with patients without systemic hypertension [16]. The complication of systemic hypertension is not a contraindication for using BV or pazopanib. Indeed, as in our case, Lou et al. reported a patient with glioblastoma (GBM) who developed PRES induced by BV, restarted BV from a low dose under good control of blood pressure, and maintained a clinical stable condition for 4 months without PRES recurrence [17]. As there are insufficient data to determine whether readministering pazopanib is safe, special care for patients is necessary. However, since the treatment options are very limited for rare cancers such as sarcoma or GBM, readministering angiogenesis inhibitors may be acceptable under appropriate management. We hope that therapeutic guidelines for PRES will contribute to the safer and more effective management of patients treated with new drugs by accumulation of more data in future.
